# Ingestion of 20 g Whey or Canola Protein Does Not Further Increase Muscle Protein Synthesis Rates During Recovery From Resistance Exercise In Healthy, Young Females

**DOI:** 10.1016/j.tjnut.2025.10.018

**Published:** 2025-10-15

**Authors:** Noortje Boot, Wesley JH Hermans, Lisa ME Kuin, Julia M Malowany, Floris K Hendriks, Ines Warnke, Joan M Senden, Alex Overman, Joy PB Goessens, Antoine Zorenc, Esther Kornips, Lex B Verdijk, Luc JC van Loon

**Affiliations:** 1 NUTRIM Institute of Nutrition and Translational Research in Metabolism, Department of Human Biology, Maastricht University Medical Centre+, Maastricht, the Netherlands; 2 dsm-firmenich, AG, Human Nutrition and Care (HNC) Innovation, R&D and Regulatory, Kaiseraugst, Switzerland

**Keywords:** native plant-based protein, fractional synthesis rate, muscle biopsy, postexercise recovery, females

## Abstract

**Background:**

Protein ingestion during recovery from exercise can further increase muscle protein synthesis rates. Plant-derived proteins are generally believed to have lesser anabolic properties than animal-derived proteins. To our knowledge, no studies have compared the impact of plant-derived with that of animal-derived protein ingestion on postexercise muscle protein synthesis rates in healthy, young females.

**Objectives:**

This study compared muscle protein synthesis rates following the ingestion of 20 g native canola protein, 20 g whey protein, or a noncaloric placebo during recovery from a single bout of resistance exercise in healthy, young females.

**Methods:**

In this randomly assigned, parallel group, double-blind placebo-controlled design, 36 healthy young (age, 23 ± 4 y; BMI, 22.7 ± 2.2 kg/m^2^) females performed 8 sets of lower-body resistance exercise at 80% of their predetermined 1-repetition maximum after which they ingested 20 g native canola protein isolate, 20 g whey protein isolate, or a noncaloric placebo. Primed continuous L-[*ring*-^13^C_6_]-phenylalanine infusions were applied with frequent sampling of blood and muscle tissue to assess postprandial plasma amino acid profiles and 5-h postexercise muscle protein synthesis rates. Data are presented as mean ± SD.

**Results:**

Plasma essential amino acid concentrations strongly increased following ingestion of both whey and canola protein when compared with the placebo treatment (peak: 2113 ± 354, 1249 ± 173, and 780 ± 60 μmol/L, respectively; *P* < 0.001) with greater postprandial plasma essential amino acid availability following whey than after canola protein ingestion (incremental AUC, 158 ± 50 and 90 ± 23 mmol/L × 5 h; *P* < 0.001). No significant differences in postexercise muscle protein synthesis rates were observed following the ingestion of whey protein isolate, canola protein isolate, and placebo (0.071 ± 0.015, 0.069 ± 0.016, and 0.061 ± 0.013%/h, respectively; treatment; *P* = 0.200).

**Conclusions:**

A single session of resistance exercise strongly increases muscle protein synthesis rates in young females. Ingestion of 20 g whey or native canola protein does not further augment muscle protein synthesis rates during the early stages of postexercise recovery in healthy, young females.

This trial was registered at clinicaltrials.gov as NCT05664269.

## Introduction

Resistance exercise stimulates muscle protein synthesis, resulting in increases in muscle mass and strength following more prolonged exercise training [1,2]. Postexercise protein ingestion further augments muscle protein synthesis rates, thereby supporting muscle conditioning [[3], [4], [5]]. Athletes are generally recommended to consume ∼20 g protein following cessation of exercise as a means to facilitate the skeletal muscle adaptive response to exercise training [6,7]. The capacity of dietary protein to stimulate postprandial muscle protein synthesis depends on both protein quantity and quality. It has been well-established that the anabolic properties of a protein source are largely determined by its digestion and absorption kinetics and amino acid composition [[8], [9], [10], [11]].

Most animal-derived proteins are considered high-quality, because they are rich in essential amino acids (EAAs) and have a well-balanced amino acid profile without apparent amino acid deficiencies [[12], [13], [14]]. Whey protein is considered the gold-standard in protein quality because it has both a high EAA content and is rapidly digested and absorbed [11,15,16]. Therefore, athletes generally prefer the consumption of whey protein or whey protein based supplements during recovery from exercise to further stimulate postexercise muscle protein synthesis rates and, as such, potentiate gains in muscle mass. Historically, plant-derived proteins are considered to have lesser anabolic properties than animal-derived proteins such as whey or milk protein [[17], [18], [19]]. The lesser anabolic properties of plant-derived proteins have been attributed to lower digestibility, lower EAA content, and deficiencies in 1 or more specific amino acids such as leucine, lysine, or methionine [[12], [13], [14]]. Therefore, plant-derived proteins are generally believed to have a lower capacity to stimulate postexercise muscle protein synthesis rates than animal-derived proteins.

Canola (*Brassica napus*) is among the highest cultivated oilseed crops in the world [20,21]. Canola press-cake is a by-product from the extraction of rapeseed oil and has a high protein content [20,22]. Currently, canola press-cake is seldom used as a food ingredient. Canola protein has a high EAA content (29%) when compared with most plant-derived proteins and compares well with other higher-quality plant protein sources such as soy (28%), pea (30%), corn (32%), and potato (37%) [14]. Furthermore, the leucine, lysine, and methionine contents in canola protein exceed their respective daily amino acid requirement for healthy adults as established by the WHO/FAO/UNU [23]. These protein characteristics are likely the reason for canola protein to be included in a protein blend that has previously been shown to increase postexercise muscle protein synthesis rates [24,25]. However, to our knowledge, no data on the muscle anabolic response following the ingestion of canola protein isolate have been reported.

Muscle protein synthesis has been extensively studied in males, leaving the female population underrepresented in exercise and nutrition research [26]. However, very few studies have assessed postexercise muscle protein synthesis rates in females [[27], [28], [29], [30], [31], [32]], studies addressing the impact of different protein sources on postexercise muscle protein synthesis rates in females are lacking. We address this gap in the literature by evaluating muscle protein synthesis rates both at rest and during postexercise recovery, with and without the ingestion of canola and whey protein isolate in an exclusive cohort of young females. Accordingly, we hypothesized that both canola and whey protein ingestion augment muscle protein synthesis rates during recovery from a single bout of resistance exercise in healthy, young females.

In the present study, we recruited 36 healthy young females to partake in a study in which we assessed the impact of protein ingestion on postexercise muscle protein synthesis rates. Contemporary stable isotope methodology, with frequent blood and muscle tissue collection, was applied to determine muscle protein synthesis rates following the ingestion of 20 g whey protein, 20 g native canola protein, or a noncaloric placebo during 5 h of recovery from a single bout of resistance exercise in healthy, young females.

## Methods

### Participants

Thirty-six healthy, young females (age, 23 ± 4 y; height, 1.69 ± 0.07 m; weight, 64.7 ± 7.6 kg) volunteered to participate in this randomized, double-blinded, parallel, placebo-controlled trial. Participants were recreationally active but did not participate in any progressive resistance exercise training program (participants’ characteristics are presented in Table 1). This study was prospectively registered at clinicaltrials.gov (NCT05664269) and was conducted between April 2023 and March 2024 at Maastricht University, the Netherlands (see Supplemental Figure 1 for the CONSORT flow diagram). All participants were informed about the purpose of the study, experimental procedures, and possible risks prior to providing informed written consent to participate. All procedures were in accordance with ethical standards of the Medical Research Ethics Committee Academic Hospital Maastricht, Maastricht University (METC22-035), and the most recent version of the Helsinki Declaration. The study was independently monitored by the Clinical Trial Center Maastricht (NL81203.068.22).

**TABLE 1:** Participant characteristics.

Parameter	Placebo (*n* = 12)	Whey (*n* = 12)	Canola (*n* = 12)
Age (y)	22 ± 4	23 ± 3	23 ± 4
Body mass (kg)	66.6 ± 3.8	62.6 ± 8.5	64.9 ± 6.9
Height (m)	1.69 ± 0.08	1.68 ± 0.06	1.70 ± 0.07
BMI (kg/m^2^)	23.4 ± 2.2	22.2 ± 2.3	22.4 ± 2.0
Bodyfat (%)	28 ± 5	24 ± 5	27 ± 4
Lean body mass (kg)	47.6 ± 3.2	45.6 ± 7.4	47.4 ± 4.2
Appendicular lean mass (kg)	22 ± 3	22 ± 4	24 ± 4
Systolic blood pressure (mm Hg)	105 ± 5	108 ± 7	112 ± 6
Diastolic blood pressure (mm Hg)	64 ± 6	66 ± 9	66 ± 7
Resting heart rate (bpm)	64 ± 8	64 ± 8	64 ± 10
1-RM leg press (kg)	192 ± 35	180 ± 44	181 ± 37
1-RM leg extension (kg)	74 ± 13	72 ± 16	77 ± 16
Hormonal contraceptive use^1^			
Naturally menstruating (no hormonal contraception)	5	10	6
Combined oral contraceptive use (e.g., Microgynon 30 and Novadien)	4	2	5
Progestin-only IUDs (e.g., Mirena and Kyleena)	2	0	1
Progestin-only implant (e.g., Implanon)	1	0	0

Values represent mean ± SD. Placebo, water; whey, 20 g whey protein isolate; canola, 20 g native canola protein isolate.

Abbreviations: IUD, intrauterine device; 1-RM, 1-repetition maximum.

1 All contraceptives belong to second-generation formulations.

### Preliminary testing

Participants aged 18–35 y, with a BMI (in kg/m^2^) between 18 and 30, underwent an initial screening to assess eligibility, whereby body height (m), body mass (kg) and blood pressure (mm Hg) were determined. Participants were deemed healthy based on their response to a medical questionnaire. Participants were excluded from participation if they reported hypertension (>140/90 mm Hg) or gastrointestinal disorders, were smoking, were taking third-generation oral contraceptives, were participating in a progressive resistance exercise training program, and/or indicated intolerance to the investigational food products. Body composition was assessed by dual-energy X-ray absorptiometry (Discovery A; Hologic). Maximal strength (1-repetition maximum) was determined on a supine leg press and seated leg extension (Technogym BV), respectively. For each exercise, subjects performed 2 warm-up sets to get familiarized with the equipment and have lifting technique corrected where necessary. Subjects then performed single repetitions at progressively increasing loads until failing to complete a valid repetition as judged by their inability to complete full range of motion on an exercise. A 2-min resting period between subsequent attempts was allowed. The screening session and experimental visit were separated by ≥3 d.

### Study design

In this randomly assigned, parallel group, double-blind placebo-controlled design, 36 young females participated in a single experimental test day during which they performed a single bout of lower-body resistance exercise after which they consumed 20 g native canola (CanolaPRO; dsm-firmenich), 20 g whey protein (Nutri Whey Isolate; FrieslandCampina), or a noncaloric placebo (*n* = 12 per group). Blood and muscle tissue were collected frequently to assess postabsorptive and postexercise muscle protein synthesis rates across intervention groups. Interventional beverage allocation (block size, *n* = 3) was according to a randomization list generated using a computerized randomizer (http://www.randomization.com/). Participants and researchers involved in performing the experimental trials and analyses were blinded to the intervention allocation.

### Diet and physical activity

The experimental trial day was scheduled within the first 10 d of the menstrual cycle for participants not taking hormonal contraceptives, which was self-reported to control for hormonal fluctuations. Participants refrained from sports, strenuous physical activities, and alcohol consumption for 3 d prior to the experimental trial day. In addition, participants reported their physical activity and dietary intake for 2 d prior to the trial day. All participants were provided the same standardized dinner meal [providing 2.5 MJ, with 50 energy (En)% carbohydrate, 29 En% fat, and 16 En% protein] the evening before the experimental trial day.

### Experimental protocol

**FIGURE 1:**
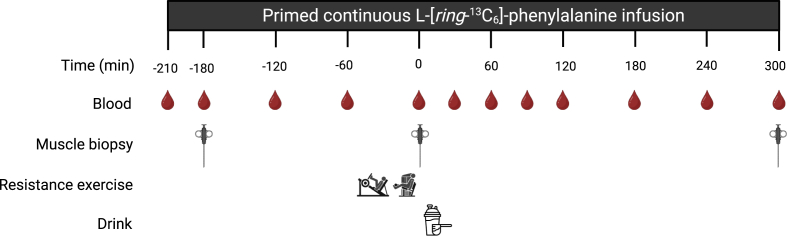
Schematic of the experimental design.

An overview of the experimental trial day is shown in Figure 1. Participants reported to the laboratory at 07:45 after an overnight fast (∼10 h). A catheter was inserted into an antecubital vein for stable isotope amino acid infusion, whereas a second catheter was inserted into a dorsal hand vein of the contralateral arm for arterialized blood sampling. Blood samples were collected at −210, −180, −120, −60, 0, 30, 60, 90, 120, 180, 240, and 300 min. To obtain arterialized blood samples, the hand was placed in a hotbox (60 °C) for 10 min prior to each blood sample collection.

After taking a baseline blood sample (*t* = −210 min), the plasma phenylalanine pool was primed with a single bolus of L-[*ring*-^13^C_6_]-phenylalanine (2.25 μmol/kg). Thereafter, a continuous intravenous infusion of L-[*ring*-^13^C_6_]-phenylalanine was initiated using a calibrated Space^plus^ Infosumat pump (B.Braun). Although resting in a supine position, arterialized blood samples were collected at −180, −120, −60, and 0 min prior to beverage ingestion. Muscle biopsy samples from the vastus lateralis were obtained at −180 and 0 min to assess postabsorptive muscle protein synthesis rates. At −45 min, the bilateral resistance exercise session was initiated, which consisted of 6 sets on both the leg press and leg extension machine. After completion of the first 2 sets as a warm-up, participants performed 4 sets of 8–10 repetitions at 80% of the predetermined 1-repetition maximum, with the last set performed until fatigue occurred. Participants rested 2 min between sets and were verbally encouraged to complete the protocol. Immediately after the exercise session, a blood sample was collected, and a second muscle biopsy (*t* = 0 min) was taken from the same leg as the first biopsy. Thereafter, the test beverage containing 20 g of protein based on the sum of total amino acids (TAAs; 23.5 g native canola or 23.4 g whey protein powder in 300 mL of water) (Table 2) was provided. For the placebo condition, only 300 mL water was provided. All beverages were flavored with 3 mL vanilla flavor (Dr. Oetker) and provided in a nontransparent shaker and consumed within 5 min. Further arterialized blood samples were collected at 30, 60, 90, 120, 180, 240, and 300 min. At 300 min, a third muscle biopsy was obtained from the vastus lateralis of the other leg to allow assessment of postprandial muscle protein synthesis rates.

**TABLE 2:** Amino acid composition of interventional drinks.

	Placebo	Whey	Canola
Alanine	0	0.9	0.8
Arginine	0	0.4	1.4
Aspartic acid	0	2.2	1.1
Cysteine	0	0.4	0.7
Glutamic acid	0	3.6	4.9
Glycine	0	0.3	0.9
Histidine	0	0.3	0.6
Isoleucine	0	1.3	0.7
Leucine	0	2.1	1.4
Lysine	0	1.9	1.3
Methionine	0	0.4	0.4
Ornithine	0	0	0
Phenylalanine	0	0.6	0.8
Proline	0	1.2	1.5
Serine	0	0.9	0.8
Threonine	0	1.4	0.7
Tyrosine	0	0.6	0.4
Valine	0	1.1	0.9
Tryptophan	0	0.4	0.3
Total EAA	0	9.6	7.3
Total NEAA	0	10.5	12.5
Total AA	0	20.1	19.7

Values for amino acid contents are in grams.

Abbreviations: EAA, essential amino acid; NEAA, nonessential amino acid; AA, amino acid.

### Protein analyses

Amino acid profiles of the protein isolates were analyzed by Eurofins, in compliance with requirements in DS EN ISO/IEC 17025 DANAK 581. In short, acid hydrolysis (ISO 13903:2005; EU 152/2009), oxidation-hydrolysis to measure cysteine and methionine (ISO 13903:2005; EU 152/2009), and alkaline hydrolysis to measure tryptophan (EU 152/2009) were applied in triplicate. Protein content was calculated based on the sum of measured amino acids. The amino acid composition of the protein isolates is presented in Table 2.

### Plasma analyses

Plasma glucose and insulin concentrations were analyzed using commercially available kits (ref. no. A11A01667; Glucose HK CP; ABX Diagnostics; and ref. no. K151BZC-3; Human Insulin Kit; Meso Scale Discovery, respectively). Plasma amino acid concentrations were determined by ultraperformance liquid chromatography-mass spectrometry (UPLC-MS; ACQUITY UPLC H-Class with QDa; Waters). Specifically, 50 μL blood plasma was deproteinized using 100 μL of 10% sulfosalicylic acid with 50 μM of MSK-A2 internal standard (Cambridge Isotope Laboratories). Subsequently, 50 μL of ultrapure demineralized water was added, and samples were centrifuged (15 min, room temperature at 21,000 × *g*). After centrifugation, 10 μL of supernatant was added to 70 μL of Borate reaction buffer (Waters). In addition, 20 μL of AccQ/Tag derivatizing reagent solution (Waters) was added after which the solution was heated to 55 °C for 10 min. Of this 100 μL derivative, 1 μL wa s injected and measured using UPLC-MS. For the determination of plasma L-[*ring*-^13^C_6_]-phenylalanine enrichments, phenylalanine was derivatized to its 6-aminoquinolyl-N-hydroxysuccinimidyl carbamate derivative, and enrichments were determined by UPLC-MS by using mass detection of masses 336, 342, and 345 for unlabeled and labeled ^13^C_6_ and ^13^C_9_ phenylalanine, respectively. Standard regression curves were applied from a series of known standard enrichment values against the measured values to assess the linearity of the mass spectrometer and to account for any isotope fractionation, which may have occurred during the analysis.

### Muscle tissue analysis

A piece of wet muscle (∼50 to 70 mg) was freeze dried for 48 h. Collagen, excessive blood, and other nonmuscle materials were subsequently removed from the muscle fibers under a light microscope. The isolated muscle fiber mass was weighed, and 35 volumes (7× wet weight of isolated muscle fibers × wet-to-dry ratio 5:1) of ice-cold 2% perchloric acid was added. Thereafter, the tissue was homogenized by sonification and centrifuged to separate the supernatant from the protein pellet. The protein pellet was washed 3 times with 1 mL of 2% perchloric acid. The amino acids were liberated from the mixed-muscle enriched protein fraction by adding 3 mL of 6 M HCl and heating it to 110 °C for 16 h. The hydrolyzed mixed-muscle protein fractions were dried under a nitrogen stream while heating to 110 °C. The dried mixed-muscle protein fraction was dissolved in a 50% acetic acid solution. The amino acids from the mixed-muscle protein fraction were passed over a Dowex exchange resin (AG 50W-X8; 100-200 mesh hydrogen form; Bio-Rad) using 2 M NH_4_OH. Subsequently, the purified amino acid solution was dried under a nitrogen stream at room temperature, followed by derivatization to their N(O,S)-ethoxycarbonyl-ethylesters. The ratio of ^13^C/^12^C of mixed-muscle protein-bound phenylalanine was determined using gas chromatography–combustion-isotope ratio mass spectrometry (Delta V; ThermoScientific) by monitoring ion masses 44, 45, and 46. Standard regression curves were applied from a series of known standard enrichment values against the measured values to assess the linearity of the mass spectrometer and to account for any isotope fractionation that may have occurred during the analysis.

### Calculations

The fractional synthetic rate (FSR; %/h) of mixed-muscle protein-enriched fractions was calculated by the standard precursor-product equation [33]:

$$FSR\;\left(\%/h\right)=\frac{\left(Eb_{2}-Eb_{1}\right)}{\left(E_{precursor}\;\cdot \;t\right)}\;\cdot 100$$

where E_*b2*_ – E_*b1*_ is the increment in mixed-muscle protein-bound L-[*ring*-^13^C_6_]-phenylalanine enrichment [expressed as mole percent excess (MPE)] between 2 muscle biopsies). E_*precursor*_ presents the weighted mean plasma tracer enrichments between consecutive blood samplings and corrected for the time between these samples, and *t* is the tracer incorporation time in hours.

Net incremental AUC (iAUC) was determined for plasma amino acid concentrations during the 5-h postexercise period, following beverage ingestion. The iAUC was calculated using the trapezoid rule, with plasma concentrations before beverage ingestion (*t* = 0 min) serving as baseline.

### Anabolic signaling and gene expression

Western blotting and mRNA analyses were performed according to protocols described in the Supplemental Methods.

### Statistical analyses

The mixed-muscle FSR over the postprandial period, comparing canola protein, whey protein, and placebo, was defined as the primary outcome measure. Secondary outcome measures included plasma glucose, insulin, amino acid concentrations, plasma iAUC, and muscle protein signaling parameters comparing the 3 different treatments. A sample size calculation was performed with differences in postprandial muscle FSR between the 3 treatments as the primary outcome. Based on previous literature comparing the ingestion of 20 g whey with placebo following exercise [4], a sample size of 12 participants per treatment including a 10% dropout rate was calculated using a power of 80% and significance level of 0.05 [34], and a difference in postexercise FSR of 0.018 %/h (or effect size 1.5). To directly assess differences in muscle protein synthesis rates between treatments over the postprandial timeframe (0–300 min), a 1-way ANOVA was performed on postprandial FSR values with treatments as between-subject factors (canola, whey, or placebo). Additionally, a 2-way repeated-measures ANOVA with time as within-subject factor and test beverage ingestion (treatment) was conducted to explore main effects and potential interactions across the postabsorptive (−180 to 0 min) as well as postprandial (0–300 min) timeframe. Plasma glucose, insulin, and amino acid concentrations; enrichments over time; and muscle protein signaling were compared between groups using a tw2-way repeated-measures ANOVA with time as within-subject factor and test beverage ingestion (treatment) as between-subject factor. In case significant time × treatment interaction was observed, separate ANOVAs were performed per time point to detect differences between treatments for each timepoint with Bonferroni-Holm post hoc corrections. Statistical analyses were performed with a software package (IBM SPSS statistics, version 27; IBM Corp). All reported *P* values were adjusted using the Bonferroni-Holm method to correct for multiple comparisons [35]. Statistical significance was set at *P* < 0.05 [34]. Data are expressed as means and SDs.

## Results

### Plasma glucose and insulin concentrations

**FIGURE 2:**
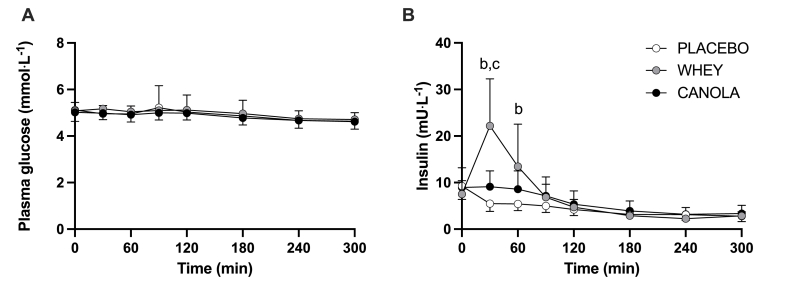
Plasma glucose (A) and insulin (B) concentrations during the 300-min postprandial period following the ingestion of native canola protein, whey protein, or placebo in healthy, young females (*n* = 12 per group). *T* = 0 min represents time of beverage intake. Canola: 20 g native canola protein isolate; whey: 20 g whey protein isolate; placebo: water. Values represent means ± SD; 2-way ANOVA with time as within-subjects variable and test beverage (treatment) as between-subjects variable; “a” denotes a significant difference (*P* < 0.05) between canola protein and placebo; “b” denotes a significant difference (*P* < 0.05) between whey protein and placebo; “c” denotes a significant difference (*P* < 0.05) between canola protein and whey protein.

Plasma glucose concentrations were not different between treatments directly prior to ingestion of the test beverages (*t* = 0 min; *P* = 0.749) (Figure 2A). After ingestion of the intervention beverages, plasma glucose concentrations decreased over time (time; *P* < 0.001) with no differences between treatments (time × treatment; *P* = 0.740). Plasma insulin concentrations (Figure 2B) significantly increased following ingestion of whey protein than that after placebo (≤*t* = 60 min; *P* < 0.001). Ingestion of canola protein did not result in significant increases in plasma insulin concentrations when compared with placebo over the full 300-min postprandial period (*P* = 0.172).

### Plasma amino acid concentrations

**FIGURE 3:**
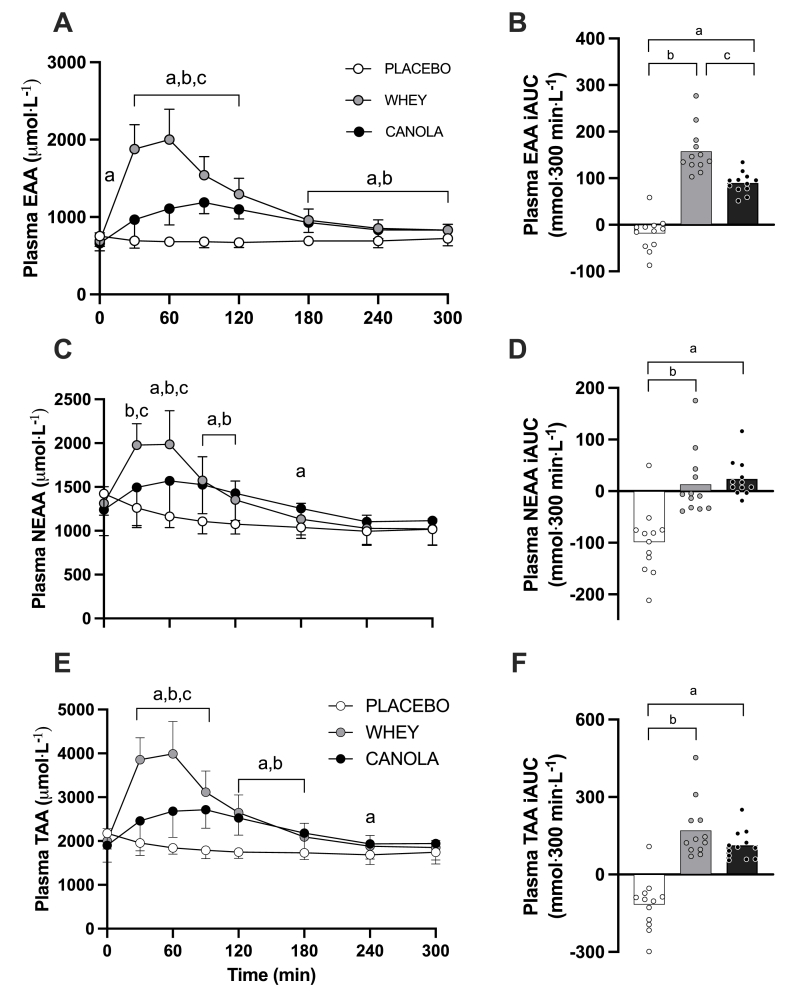
Postprandial plasma EAA (A), NEAA (C), and TAA (E) concentrations during the 300-min postprandial period and their incremental area under the curve (iAUC; panels B, D and F, respectively) following ingestion of canola protein, whey protein, and placebo in healthy, young females (*n* = 12 per group). *T* = 0 min represents time of beverage intake. Canola, 20 g native canola protein isolate; whey, 20 g whey protein isolate; placebo, water. Values represent means ± SD; 2-way ANOVA with time as within-subjects variable and test beverage (treatment) as between-subjects variable; “a” denotes a significant difference (*P* < 0.05) between canola protein and placebo; “b” denotes a significant difference (*P* < 0.05) between whey protein and placebo; “c” denotes a significant difference (*P* < 0.05) between canola protein and whey protein. EAA, essential amino acid; NEAA, nonessential amino acid; TAA, total amino acid.

Plasma EAA, nonessential amino acid (NEAA), and TAA concentrations are presented in Figure 3. Plasma EAA concentrations strongly increased following protein ingestion (Figure 3A) (time; *P* < 0.001), with less of an increase following canola compared with that after whey protein ingestion (time × treatment; *P* < 0.001). Consequently, canola protein ingestion resulted in lower peak plasma EAA concentrations than whey protein ingestion (1248 ± 173 and 2113 ± 354 μmol/L, respectivley; *P* < 0.001). In addition, the time to reach these peak concentrations was also longer following canola than that after whey protein ingestion (90 ± 36 and 53 ± 19 min, respectively; *P* < 0.001). The overall increase in EAAs over the entire 300 min postprandial period, expressed as iAUC, was 43% less following canola compared with that after whey protein ingestion (Figure 3B) (*P* < 0.001). Ingestion of both canola and whey protein resulted in greater EAA availability than placebo ingestion (both *P* < 0.001). Plasma NEAA concentrations increased following protein ingestion (Figure 3C) (time; *P* < 0.001), with less of an increase following canola than that after whey protein ingestion (time × treatment; *P* < 0.001). Canola protein ingestion resulted in lower peak plasma NEAA concentrations than whey protein ingestion (1675 ± 395 and 2092 ± 357 μmol/L, respectively; *P* < 0.001). In addition, the time to reach these peak concentrations was also longer following canola than that after whey protein ingestion (75 ± 41 and 43 ± 20 min, respectively; *P* < 0.001). The overall increase in NEAAs over the entire 300-min postprandial period, expressed as iAUC, that was not different between canola protein and whey protein (Figure 3D) (*P* = 0.656) but both canola and whey protein showed significantly greater values than placebo (both *P* < 0.001). Overall, plasma TAA concentrations increased following protein ingestion (Figure 3E) (time; *P* < 0.001), with a lesser increase following canola ingestion than that after whey protein ingestion (time × treatment; *P* < 0.001). Overall, TAA availability (iAUC) over the entire 300-min postprandial period was greater for both canola and whey protein than that for placebo (Figure 3F) (both *P* < 0.001), with no differences between protein sources (*P* = 0.137).

**FIGURE 4:**
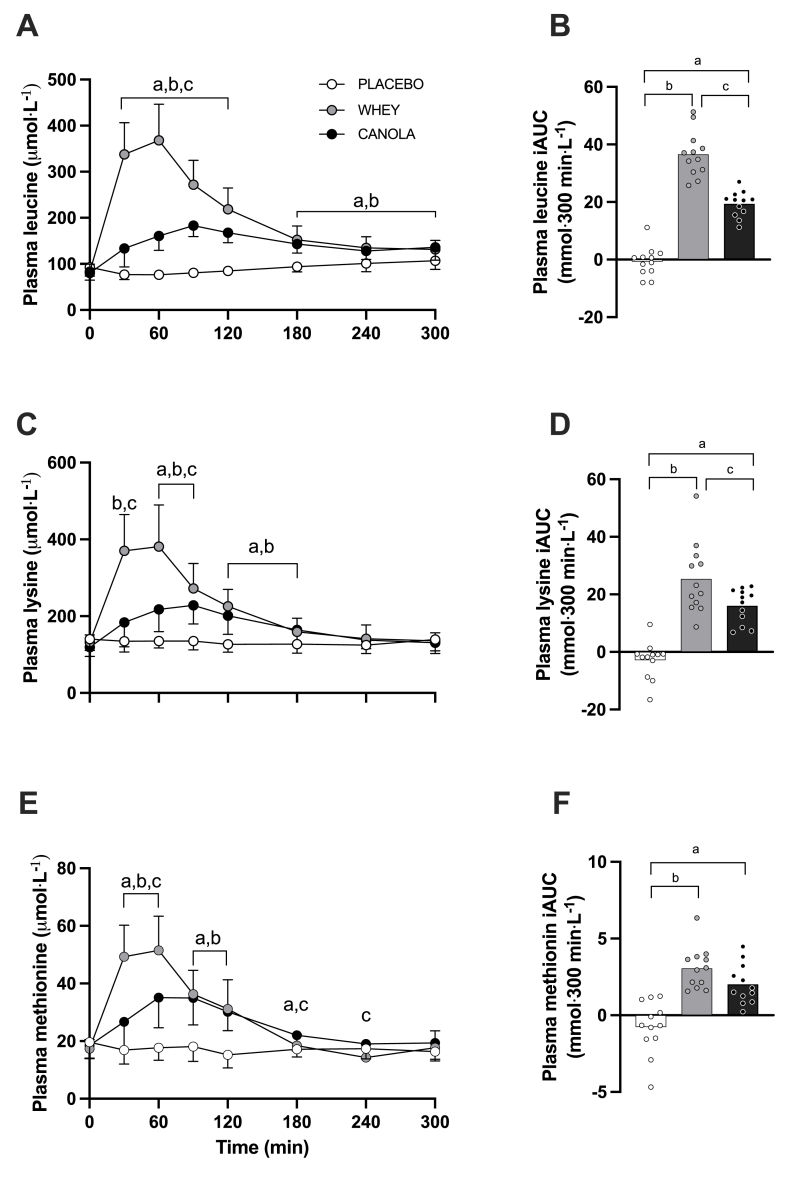
Postprandial plasma leucine (A), lysine (C), and methionine (E) concentrations during the 300-min postprandial period and their incremental area under the curve (iAUC; panels B, D, and F) following ingestion of canola protein, whey protein, and placebo in healthy young females (*n* = 12 per group). *T* = 0 min represents time of beverage intake. Canola: 20 g native canola protein isolate; Whey: 20 g whey protein isolate, Placebo: water. Values represent means ± SD; two-way ANOVA with time as within-subjects variable and test beverage (treatment) as between-subjects variable. “a” denotes a significant difference (*P* < 0.05) between canola protein and placebo; “b” denotes a significant difference (*P* < 0.05) between whey protein and placebo; “c” denotes a significant difference (*P* < 0.05) between canola protein and whey protein.

Plasma leucine concentrations increased over time following protein ingestion (Figure 4A) (time; *P* < 0.001). Ingestion of canola protein resulted in lower peak plasma leucine concentrations than whey protein ingestion (192 ± 26 and 398 ± 67 μmol/L, respectively; *P* < 0.001). The time to reach peak plasma leucine concentrations was longer following canola than that after whey protein ingestion (90 ± 36 and 53 ± 19 min, respectively; *P* < 0.001). Protein ingestion resulted in increased plasma leucine availability, expressed as iAUC, when compared with placebo (both *P* < 0.001), but lower following canola than that after whey protein ingestion (*P* < 0.001).

Plasma lysine concentrations increased over time following protein ingestion (Figure 4C) (time; *P* < 0.001). Ingestion of canola protein resulted in lower peak plasma lysine concentrations than whey protein ingestion (245 ± 56 and 415 ± 98 μmol/L, respectively; *P* < 0.001). The time to reach peak lysine concentrations was significantly longer following canola than that after whey protein ingestion (88 ± 37 and 45 ± 20 min, respectively; *P* < 0.001). The overall increase in plasma lysine availability was 37% less following canola than that after whey protein ingestion (*P* = 0.014). Plasma methionine concentrations increased over time following protein ingestion (Figure 4E) (time; *P* < 0.001). Ingestion of canola protein resulted in lower plasma methionine concentrations than whey protein ingestion (39 ± 9 compared with 56 and 12 μmol/L, respectively; *P* < 0.001). The time to reach these peak concentrations was longer following canola protein than that after whey protein ingestion (83 ± 19 and 53 ± 19 min, respectively; *P* < 0.001). The overall plasma methionine availability was greater following protein ingestion than that after placebo (both *P* < 0.001), with no differences between canola and whey protein ingestion (*P* = 0.094).

In general, increases in plasma amino acid concentrations revealed significant differences over time between canola protein and whey protein compared with placebo (time × treatment; all *P* < 0.05) for all other measured amino acids. The increases in plasma amino acid concentrations over the entire 300-min postprandial period (iAUC) were significantly greater (Supplemental Figure 2) (all *P* < 0.05) when canola protein was compared with placebo (except for glutamic acid) and whey protein with placebo (except for glutamic acid and glycine).

### Plasma and muscle L-[*ring*-^13^C_6_]-phenylalanine enrichments

**FIGURE 5:**
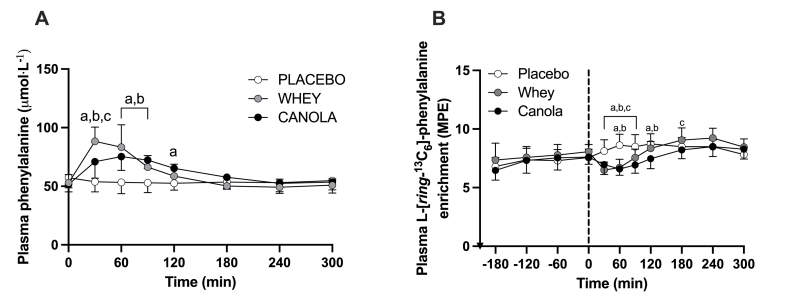
Postprandial plasma phenylalanine concentrations (A) and plasma L-[*ring-*^13^C_6_]*-*phenylalanine enrichments (B) during the complete infusion protocol in healthy young females (*n* = 12 per group). *T* = 0 min represents time of beverage intake. Canola, 20 g native canola protein isolate; whey, 20 g whey protein isolate; placebo, water. Values represent means ± SD; 2-way ANOVA with time as within-subjects variable and test beverage (treatment) as between-subjects variable; “a” denotes a significant difference (*P* < 0.05) between canola protein and placebo; “b” denotes a significant difference (*P* < 0.05) between whey protein and placebo; “c” denotes a significant difference (*P* < 0.05) between canola protein and whey protein.

Plasma phenylalanine concentrations and L-[*ring-*^13^C_6_]-phenylalanine enrichments over time are presented in Figure 5A and B, respectively. Plasma phenylalanine concentrations increased significantly over time following protein ingestion (Time; *P* = 0.001). The increase in plasma phenylalanine concentrations differed significantly between canola and whey protein but were both greater than that of the placebo condition (time × treatment; *P* < 0.001). Plasma L-[*ring-*^13^C_6_]-phenylalanine enrichments throughout the basal period (*t* = −210 until *t* = 0 min) were not different between treatments and averaged 7.23 ± 0.53, 7.70 ± 0.31, and 7.26 ± 0.32 MPE for canola protein, whey protein, and placebo treatment, respectively (time × treatment; *P* = 0.259). Following the ingestion of the interventional drinks, plasma L-[*ring-*^13^C_6_]-phenylalanine showed a transient decline in enrichments in the protein treatments when compared with the placebo treatment (time × treatment; *P* < 0.001). During the entire postprandial period, mean plasma L-[*ring-*^13^C_6_]-phenylalanine enrichments averaged 7.98 ± 1.09, 7.57 ± 0.77, and 8.40 ± 0.32 MPE for whey protein, canola protein, and placebo treatments, respectively (treatment; *P* = 0.042). Mixed-muscle protein-bound L-[*ring-*^13^C_6_]-phenylalanine enrichments increased over time from 0.0085 ± 0.0039, 0.0096 ± 0.0037, and 0.0090 ± 0.0030 MPE at *t* = 0 min to 0.0380 ± 0.0083, 0.0398 ± 0.0085, and 0.0360 ± 0.0078 MPE at *t* = 300 min in the canola protein, whey protein, and placebo treatment, respectively (time; *P* < 0.001). No differences in mixed-muscle protein-bound L-[*ring-*^13^C_6_]-phenylalanine enrichments were observed between treatment groups over the 5-h postprandial period (time × treatment; *P* = 0.463).

### Muscle protein synthesis rates

**FIGURE 6:**
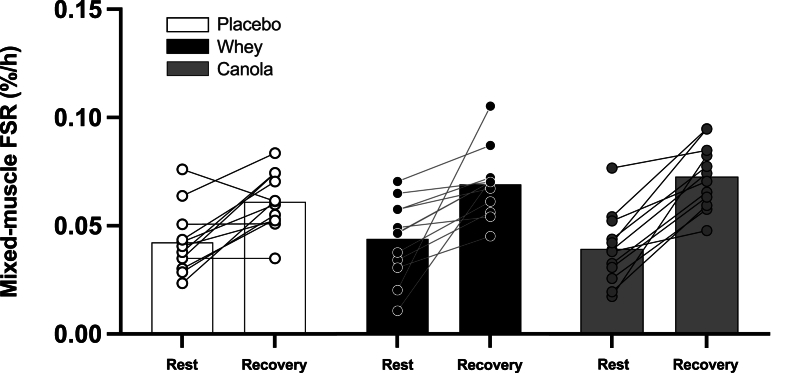
Mixed-muscle protein fractional synthesis rates (FSR) at different periods prior to (rest) and following ingestion of test beverages (recovery) in healthy young females (*n* = 12 per group). Canola, 20 g native canola protein isolate; whey, 20 g whey protein isolate; placebo, water. Values represent means ± SD; 2-way ANOVA with time as within-subjects variable and test beverage (treatment) as between-subjects variable. ∗A significant difference from basal values (*P* < 0.05).

Muscle protein synthesis rates (expressed as FSR) calculated based on the plasma precursor pool are presented in Figure 6. Muscle protein synthesis rates prior to ingestion of the intervention drinks averaged 0.038 ± 0.016, 0.044 ± 0.018, and 0.042 ± 0.015 %/h for the canola protein, whey protein, and placebo treatment, respectively, with no significant differences between treatments [F(2,33) = 0.345; *P* = 0.711; *η*^2^*p* = 0.020]. Postexercise muscle protein synthesis rates increased significantly to 0.071 ± 0.015, 0.069 ± 0.016, and 0.061 ± 0.013 %/h for canola protein, whey protein, and placebo treatment, respectively (time; *P* < 0.001). No significant differences in postexercise muscle protein synthesis rates were observed between treatments throughout the 5-h recovery period [F(2,33) =1.689, *η*^2^*p* = 0.093; treatment; *P* = 0.200].

### Anabolic signaling

The phosphorylation status (ratio of phosphorylated to total protein) of key proteins involved in the initiation of muscle protein synthesis are shown in Supplemental Figure 3. For all measured proteins, no significant treatment, nor time × treatment interactions, were observed. No significant changes were observed for muscle mTOR (Ser2448) and p70S6K (Thr389) phosphorylation status between 0 min (following resistance exercise) and 300 min (5 h after beverage ingestion; time—*P* = 0.536 and *P* = 0.887, respectively) (Supplemental Figure 3A, B). When compared with basal levels, the phosphorylation status of p70S6K (Thr421/Ser424) and rpS6 (Ser240/244) increased immediately following resistance exercise (*t* = 0 min) but returned to baseline values following the 300-min postprandial period (Supplemental Figure 3C, E) (time; *P* < 0.001). The phosphorylation status of muscle rpS6 (Ser 235/236) increased immediately following resistance exercise (*t* = 0 min) and sustained throughout the 300-min postprandial period when compared with basal levels (Supplemental Figure 3D) (time; *P* < 0.001). The phosphorylation status of muscle 4E-BP1 (Thr37/46) decreased immediately following resistance exercise (*t* = 0 min) but was significantly greater at the end of the 300-min postprandial period than basal values (Supplemental Figure 3F) (time; *P* < 0.001).

### Gene expression

Skeletal muscle mRNA expression for selected genes involved in muscle proteolysis are shown in Supplemental Figure 4. No treatment effects nor time × treatment interactions were observed for muscle FOXO1 (Supplemental Figure 4A), MuRF1 (Supplemental Figure 4B), and MaFbx (Supplemental Figure 4C). When compared with basal levels (*t* = −180 min), mRNA expression of FOXO1 increased immediately following resistance exercise (*t* = 0 min) and remained elevated throughout the the 300-min postprandial period (time; *P* < 0.001). Muscle mRNA expression of MuRF1 increased immediately following resistance exercise (*t* = 0 min) but returned to basal levels following the 300-min postprandial period (time; *P* = 0.005) (Supplemental Figure 4B). Muscle mRNA expression of MaFbx was significantly lower at the 300-min time point than that at basal levels (*t* = −180 min) and immediately following resistance exercise (*t* = 0 min; time; *P* < 0.001) (Supplemental Figure 4C).

## Discussion

In the present study, we observed a substantial increase in muscle protein synthesis rates following a single bout of resistance exercise in healthy, young females. Ingestion of 20 g whey or native canola protein strongly increased plasma amino acid concentrations throughout the 5 h of postexercise recovery but did not further augment muscle protein synthesis rates when compared with those after consuming a noncaloric placebo.

Following an overnight fast, we applied continuous L-[*ring-*^13^C_6_]-phenylalanine infusions to first assess basal, postabsorptive muscle protein synthesis rates in these 36 healthy, young females. Fasting muscle protein synthesis rates averaged 0.041 ± 0.016 %/h (Figure 6). These data are in line with previously published data on resting muscle protein synthesis rates, whcih are mainly acquired in healthy, young males [31,[36], [37], [38], [39], [40]]. Following baseline measurements, all subjects performed a single bout of strenuous resistance exercise. The exercise protocol was well-tolerated despite the fact that these recreationally active, female volunteers did not routinely perform resistance exercise. During subsequent postexercise recovery, muscle protein synthesis rates significantly increased by >50% during the 5-h recovery period in the placebo treatment (0.061 ± 0.013 %/h) (Figure 6). The exercise-induced increases in muscle protein synthesis rates in these healthy females seem to be comparable with previously acquired data acquired in healthy, young males [31,[36], [37], [38], [39], [40]].

Athletes generally consume protein supplements or protein-rich foods during recovery from resistance exercise as a means to increase postexercise amino acid availability and, as such, to further augment postexercise muscle protein synthesis rates [41]. Following the ingestion of 20 g whey protein we observed a rapid rise in circulating plasma amino acid concentrations (Figure 3), with plasma leucine concentrations reaching peak valuess well within 60 min after protein ingestion (338 ± 13 μmol/L) (Figure 4A). Plant-derived proteins are generally less rapidly digested and absorbed and contain less leucine. In the present study, we compared plasma amino acid profiles following ingestion of canola with those after whey protein ingestion. We selected canola protein because, unlike many other plant-derived proteins, canola protein does not have deficiencies in specific EAAs according to the WHO/FAO/UNU requirements [23]. This approach allowed us to isolate the effect of different protein sources without the presence of specific amino acid deficiencies as confounding variables and to examine whether muscle protein synthesis rates differ following the ingestion of such a complete plant-derived protein when compared with whey protein as the reference. Still, differences in amino acid composition between canola and whey protein were reflected in the overall postprandial plasma amino acid responses (FIGURE 3, FIGURE 4). Ingestion of 20 g canola protein resulted in an attenuated rise in circulating plasma EAAs (Figure 3A), with postprandial plasma amino acid availability being substantially less (∼43%) when compared with that after whey protein (Figure 3B). These findings show similarities to previous reports of an attenuated rise in circulating amino acids following the ingestion of various plant-derived proteins, such as soy [17,18], wheat [10], potato [36], corn [42], and pea [43] protein when compared with the ingestion of equivalent doses of animal-derived proteins. The attenuated plasma amino acid response following ingestion of plant- or animal-derived proteins may be attributed to differences in protein structure and function and accompanying antinutritional factors present in plant-derived proteins that compromise protein digestion and amino acid absorption and/or stimulate amino acid retention in splanchnic tissues [[44], [45], [46], [47]]. Collectively, the present data show that despite its complete amino acid composition, ingestion of canola protein displays an attenuated rise in plasma amino acid availability when compared with the ingestion of an equivalent amount of whey protein.

Protein ingestion during recovery has been shown to augment postexercise muscle protein synthesis rates [48,49]. In this study, we assessed the impact of canola and whey protein on postexercise muscle protein synthesis rates. Previous work suggests that postprandial amino acid availability may be predictive of the anabolic response following protein ingestion [15,17,50]. Despite the attenuated postprandial rise in amino acid availability following canola when compared with that after whey protein ingestion (Figure 3D, E), we failed to detect differences in postprandial muscle protein synthesis rates following ingestion of both proteins. From the pre-exrecise to postexercise recovery period, muscle protein synthesis rates increased by ∼85% following canola protein and by ∼ 58% following whey protein ingestion (Figure 6). The increase in muscle protein synthesis rates following protein ingestion were not significantly different from the placebo condition (∼44%) (Supplemental Data). The absence of a stimulating effect of ingesting 20 g protein on postexercise muscle protein synthesis rates is in line with some [[51], [52], [53]] but certainly not all [4,27,36,49,50,[54], [55], [56], [57], [58]] studies. Notably, most of these studies assessing muscle protein synthesis rates following a single session of resistance exercise were conducted in males, or in mixed-sex cohorts, but seldom in females exclusively. Although we did not observe differences in postprandial muscle protein synthesis rates following the ingestion of both protein sources on muscle protein synthesis rates, these findings should be interpreted with caution. The absence of a detectable effect of protein ingestion on muscle protein synthesis may relate to various factors such as the intensity of exercise, the duration of the recovery period assessed, and the variability in the exercise training response in young females. Because there was no apparent explanation for the absence of a greater increase in postexercise muscle protein synthesis following protein ingestion, we also explored whether the molecular signaling responses of key proteins regulating protein translation-initiation could offer mechanistic insights (Supplemental Figure 3). Resistance exercise increased the phosphorylation status of p70S6K (Thr421/Ser424), rpS6 (Ser235/236), rpS6 (Ser 240/244), and 4E-BP1 (Thr37/46), after which it remained elevated throughout the subsequent 5-h postprandial period. In line with the absence of differences in postprandial muscle protein synthesis rates, we observed no differences in the anabolic signaling responses between treatments. Although our biopsy timepoints accurately capture the sustained muscle protein synthetic response to exercise and protein ingestion (0–300 min), it should be noted that anabolic signaling was not considered the primary outcome of the present study. With muscle tissue collection at 5 h following protein ingestion, transient peak signaling events that typically occur in the first 2 h following protein ingestion will be missed.

In the present study, we included young females who were recreationally active but not routinely involved in a progressive resistance exercise training. Because exercise induced a very strong anabolic stimulus across all 3 treatments (Figure 6), we speculate that the intensity of the exercise bout was of such a magnitude that the impact of additional protein ingestion was negligible during the early stages of postexercise recovery. In agreement, Apicella et al. [59] previously reported no differences in postexercise muscle protein synthesis rates following the ingestion of various doses of amino acids in young females. In the present study, exercise-induced proteolysis, indicated by the expression of the genes *FOXO1*, *MuRF1*, and *MaFbx* (Supplemental Figure 4), may have provided the release of sufficient amounts of amino acids to support the postexercise increase in muscle protein synthesis rates, thereby mitigating the proposed stimulating effect of ingesting additional protein. Importantly, the current literature does not provide data on postabsorptive and postexercise muscle protein synthesis rates with and without protein ingestion in healthy, young females. In this study, we showed a robust increase in muscle protein synthesis rates during recovery from a single bout of resistance exercise in healthy, young females. Interestingly, this robust response was not further increased by the ingestion of a single bolus of canola or whey protein isolate in these healthy, young females. More work will be required to assess whether the impact of postexercise protein ingestion on (acutely measured) muscle protein synthesis rates is more modest, or even absent, in women when compared with that of men.

Although PubMed provides >90 publications in which the acute postexercise muscle protein synthesis responses have been assessed in young adults, only 13 of these studies have also included females, with only 2 studies having included females only [58,59]. This underlines the need to perform more exercise and nutrition studies in females, as is increasingly being recognized [26,58]. Although sex-related differences may be responsible for the apparent discrepancy from the existing literature, we have previously shown a robust postprandial muscle protein synthetic response in females after consuming 25 g whey protein at rest [60]. Consequently, we speculate that the absence of a difference in postexercise muscle protein synthesis rates between the placebo and the protein treatments in this study is likely attributed to a more pronounced exercise response in these untrained females. Furthermore, it could be speculated that a greater amount of protein could be required to trigger a more robust postprandial response following exercise. However, it should be noted that the protein dose provided in this study translates in a relative protein intake of 0.31 g/kg body mass and 0.44 g/kg lean body mass respectively (Table 1). These relative doses exceed previously suggested doses to maximally stimulate muscle protein synthesis in healthy, young males (0.24 g/kg body mass and 0.25 g/kg lean body mass) [6]. Interestingly, recent work by Mallinson et al. [58] in trained young females demonstrated that ingestion of 0.22 g protein/kg body mass did not significantly stimulate muscle protein synthesis rates over a 4-h postexercise period, whereas 0.46 g protein/kg body mass appeared effective. Combined with those of Mallinson et al. [58], our data indicate that there may be a critical protein dose (somewhere between 0.31 and 0.46 g/kg body mass) that is required to effectively stimulate muscle protein synthesis rates in healthy young females. The present work focused exclusively on healthy, young females with a design chosen to provide sex-specific data that contribute to a more inclusive understanding of muscle protein metabolism. Rather than aiming for direct sex-based comparisons, which are often confounded by differences in body composition, protein requirements, and exercise responses, we argue for the value of well-controlled, sex-specific studies. We acknowledge that our reliance on self-reported menstrual cycle phase, rather than biochemical verification through hormonal assessment, deviates from best-practice methods for hormonal characterization [61]. As such, whether the present data imply that females may respond differently to the ingestion of (different doses of) protein during recovery from exercise will warrant more research, including clear verification and reporting of hormonal fluctuations.

In conclusion, a single session of resistance exercise strongly increases muscle protein synthesis rates in healthy, young females. Ingestion of 20 g canola protein during recovery from exercise is followed by an attenuated postprandial rise in circulating amino acids when compared with the ingestion of an equivalent amount of whey protein. Ingestion of 20 g canola or whey protein during recovery from a single bout of exercise does not further increase muscle protein synthesis rates in healthy, young females.

## Author contributions

The author’s responsibilities were as follows – LJCvL, LBV, WJHH, NB: designed the research; NB, WJHH, LMEK, FKH, JMS AO, JPBG, AZ, EK, LBV, LJCvL: conducted the research, NB, WJHH, LB, LJCvL: analyzed the data, NB, LJCvL wrote the paper; NB, WJHH, IW, LBV, LJCvL: had primary responsibility for final content; and all authors: read and approved the final manuscript.

## Data availability

Data are available on request.

## Funding

The presented research was co-funded by dsm-firmenich AG, Delft, Netherlands. The researchers are responsible for the study design, data collection and analyses, decision to publish, and preparation of the manuscript. The industrial partners have contributed to the project through regular discussion and were involved in the study design,—more specifically, the choice of interventional products—which were produced by these sponsors. The funder had no role in data collection and analysis, decision to publish, or preparation of the manuscript.

## Conflicts of interest

LJCvL reports financial support and equipment, drugs, or supplies were provided by dsmfirmenich. IW reports a relationship with dsm-firmenich that includes: employment. LJCvL and LBV have received research grants, consulting fees, speaking honoraria, or a combination of these for research on the impact of exercise and nutrition on muscle metabolism. A full overview on research funding is provided at: https://www.maastrichtuniversity.nl/l.vanloon. All other authors report no conflicts of interest.
