# Supplementary material for: Ingestion of 20 g Whey or Canola Protein Does Not Further Increase Muscle Protein Synthesis Rates During Recovery From Resistance Exercise In Healthy, Young Females
Source: J Nutr. 2025 Oct 15;155(12):4123–35. doi: 10.1016/j.tjnut.2025.10.018 (PMC12799512; doi:10.1016/j.tjnut.2025.10.018)
Supplement: Multimedia component 1 [file mmc1.doc]

**SUPPLEMENTAL MATERIAL BELONGING TO:**

**Ingestion of 20 g whey or canola protein does not further increase muscle protein synthesis rates during recovery from resistance exercise in healthy, young females**

Noortje Boot^1^, Wesley J.H. Hermans^1^, Lisa M.E. Kuin^1^, Julia M. Malowany^1^, Floris K. Hendriks^1^, Ines Warnke^2^, Joan M. Senden^1^, Alex Overman^1^, Joy P.B. Goessens^1^, Antoine Zorenc^1^, Esther Kornips^1^, Lex B. Verdijk^1^, Luc J.C. van Loon^1^

*^1^ NUTRIM Institute of Nutrition and Translational Research in Metabolism, Department of Human Biology, Maastricht University Medical Centre+, Maastricht, The Netherlands*

*^2^ dsm-firmenich, AG, Human Nutrition and Care (HNC) Innovation, R&D and Regulatory, Kaiseraugst, Switzerland*

**SUPPLEMENTARY METHODS**

**Western blotting**

A portion of each muscle sample frozen for biochemical analyses was homogenized in isolation buffer consisting of 2.29 g sucrose, 0.606 g Tris, 0.373 g KCl and 0.372 g EDTA in 100 ml of milliQ water, final pH 7.4 supplemented with 1 tablet Complete mini (11836153001; Roche) and 1 tablet PhosStop (04906837001; Roche) per 10 mL of buffer. After homogenization, each muscle extract was centrifuged for 20 min at 10.000*g* (4^0^C) and sample buffer was added to the supernatant to final concentrations of 60 mM Tris, 10% glycerol, 20 mg/mL SDS, 0.1 mM dithiothreitol, 20 μg/mL bromophenol blue. The supernatant was then heated for 5 min at 100^0^C and immediately placed on ice. Immediately before analyses, the muscle extraction sample was centrifuged for 1 min at 1000*g* at room temperature (RT). The amount of sample loaded on the gel was based on total protein content and was the same for every sample. Following a Bradford assay, 30 μg protein was loaded in each lane. Protein samples were run on a Criterion TGX Stain-free 4-15% gel (Order No. 567-8085; Bio-Rad) ± 75 min at 150 V (constant voltage) and transferred onto a Trans-blot Turbo 0.2 μm nitrocellulose membrane (Order No. 170-4159; Bio-Rad) in 10 min at 2.5 A and 25 V. Specific proteins were detected by overnight incubation at 4^0^C on a shaker with specific antibodies in 0.1% Tween 20-PBS, after blocking for 60 min at RT in 50% PBS/ Odyssey blocking buffer. Primary phospho-specific antibodies, anti-phospho-mTOR (Ser^2448^), anti-phospho-S6K1(Thr^389^), anti-phospho-S6K1(Thr^421^/ Ser^424^), anti-phospho-rS6 (Ser^240^/Ser^244^), anti-phospho-rS6 (Ser^235/236^) and anti-phospho-4EBP1 (Thr^37^/^46^) were purchased from Cell Signaling Technology (Danvers, MA, USA). After quantification of the phospho signal, membranes were stripped in 2.5X LiCor Newblot Nitro Stripping buffer (928-40030, LiCor BioSciences) for 20 min at RT/37°C. Membranes then were incubated with their non-phospho counterpart antibodies. In addition, anti-mTOR, anti-p70 S6K1, anti-rS6, and anti-4E-BP1 were also purchased from Cell Signaling Technology. Following incubation, membranes were washed three times 10 min in 0.1 % PBS-Tween 20. Next, samples were incubated on a shaker (1 h at RT) with infrared secondary antibodies, donkey anti-rabbit IRDYE 800CW (dilution 1:10000; Cat. No. 926-32213; Li-Cor Biosciences) and donkey anti-mouse IRDYE 680RD (dilution 1:5000; Cat. No. 926-68072; Li-Cor Biosciences) dissolved in 0.1% Tween, 20-PBS. After a final wash step (3 x 10 min) in 0.1 % Tween 20-PBS and once 10 min in PBS, protein quantification was performed by scanning on an Odyssey Infrared Imaging System (Li-Cor, Biotechnology, Lincoln, NE). Phosphorylation status as a proxy of activation of the signaling proteins was expressed relative to the total amount of each protein.

**mRNA analyses**

mRNA analysis was performed on muscle tissue (t = -180, 0, and 300 min). Total RNA was isolated from 10–20 mg of frozen muscle tissue using TRIzol® Reagent (Life Technologies, Invitrogen, Carlsbad, CA, USA), in accordance with the manufacturer's instructions. Total RNA quantification was carried out spectrophotometrically at 260 nm (NanoDrop ND-1000 Spectrophotometer; Thermo Fisher Scientific) and RNA purity was determined as the ratio of readings at 260/280 nm. Thereafter, first strand cDNA was synthesized from 1 µg of RNA sample using iScript™ cDNA synthesis kit (Bio-Rad; Cat. No. 170–8891). Taqman^TM^ PCR was carried out using a 7300 Real Time PCR System (Applied Biosystems, Foster City, CA, USA), with 2 µL of cDNA, 12.5 µL of Taqman™ master mix, 1.25 µL of Taqman™ probe and 9.25 µL of H2O in a 25 µL final well volume. Each sample was run in duplicate together with a serial dilution standard curve. The housekeeping gene 18S was used as an internal control. Taqman^TM^ primer/probe sets were obtained from Applied Biosystems for the following genes: FOXO1 (Hs 0 105 4576_m1), MuRF1 (Hs 002 61590_m1), and MAFbx (Hs 0 104 1408_m1). The thermal cycling conditions used were: 2 min at 50°C, 10 min at 95°C, followed by 40 cycles at 95°C for 15 s and 60°C for 1 min. Ct values of the target genes were normalized to Ct values of the internal control and final results were calculated as relative expression against the standard curve.

**SUPPLEMENTARY FIGURES**

**Supplemental Figure 1.** Post-prandial plasma amino acid concentrations during the 300 min post-prandial period following the ingestion of native canola *vs* whey *vs* placebo. Time = 0 min represents time of beverage intake. Panels B, D, F, H, J, L, N, P, R, T, V, X, Z, AB, AD represent the 0 – 5 h incremental area under curve (iAUC) following ingestion of the test beverages. Canola: 20 g native canola protein isolate, Whey: 20 g whey protein isolate, Placebo: water. ‘’a’’ denotes a significant difference (*P<0.05*) between native canola protein *vs* placebo, ‘’b’’ denotes a significant difference (*P<0.05*) between whey protein *vs* placebo, ‘’c’’ denotes a significant difference (*P<0.05*) between native canola protein *vs* whey protein. Analyzed with repeated measures ANOVA with time as within-subject variable and test beverage (treatment) as between-subject variable. Post-hoc Bonferroni-Holm corrections were used to determine differences between groups.

**
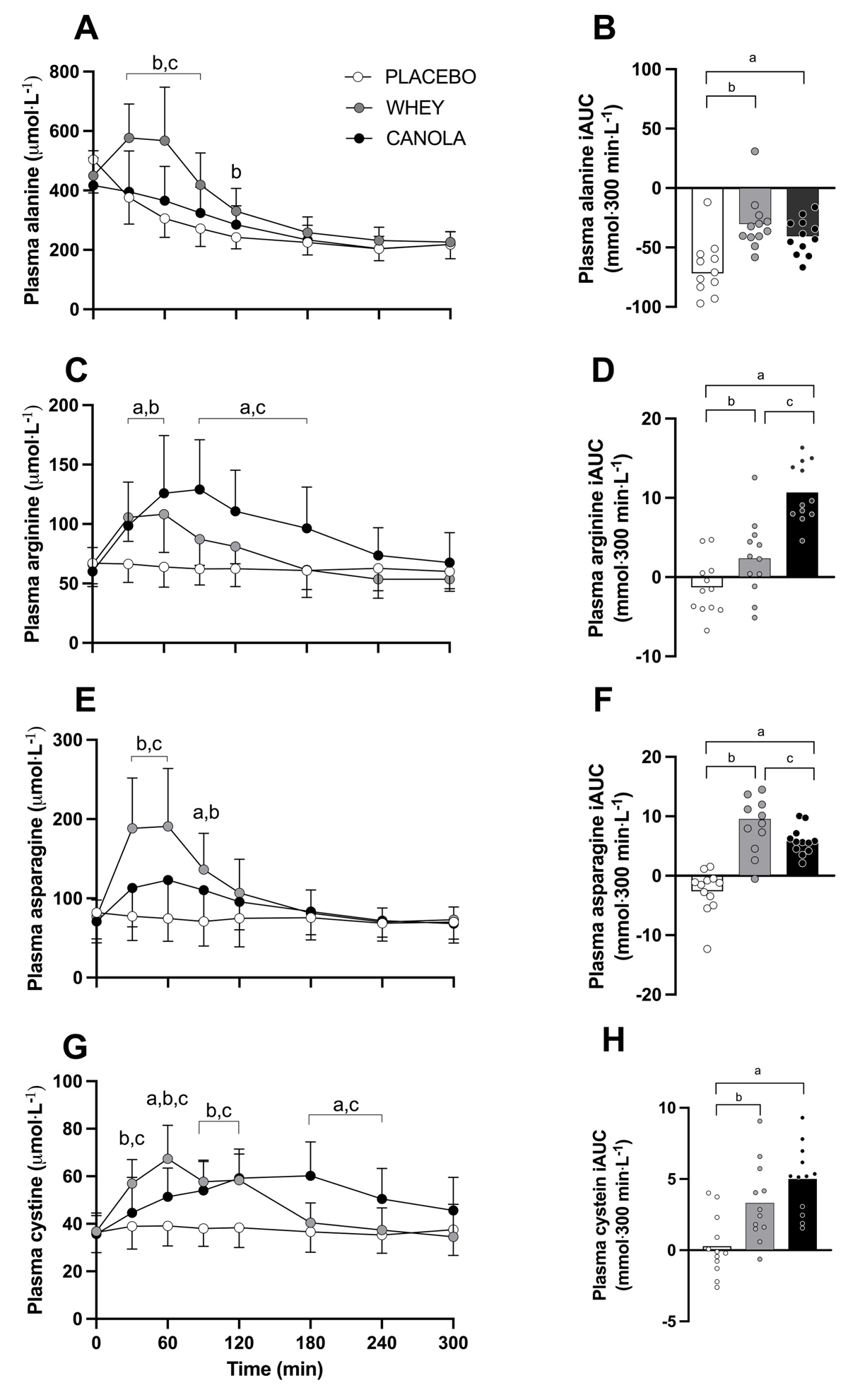
**

**
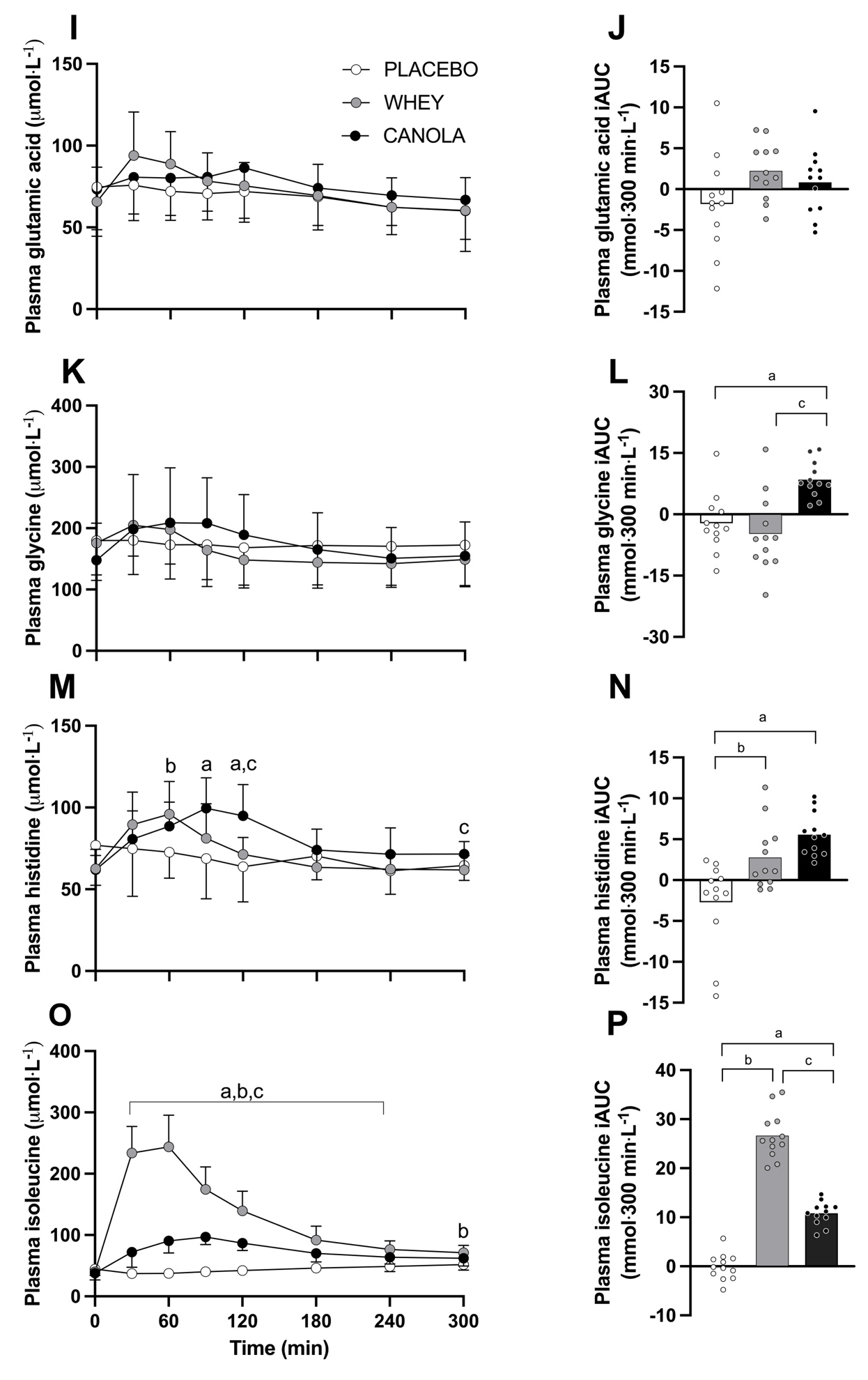
**


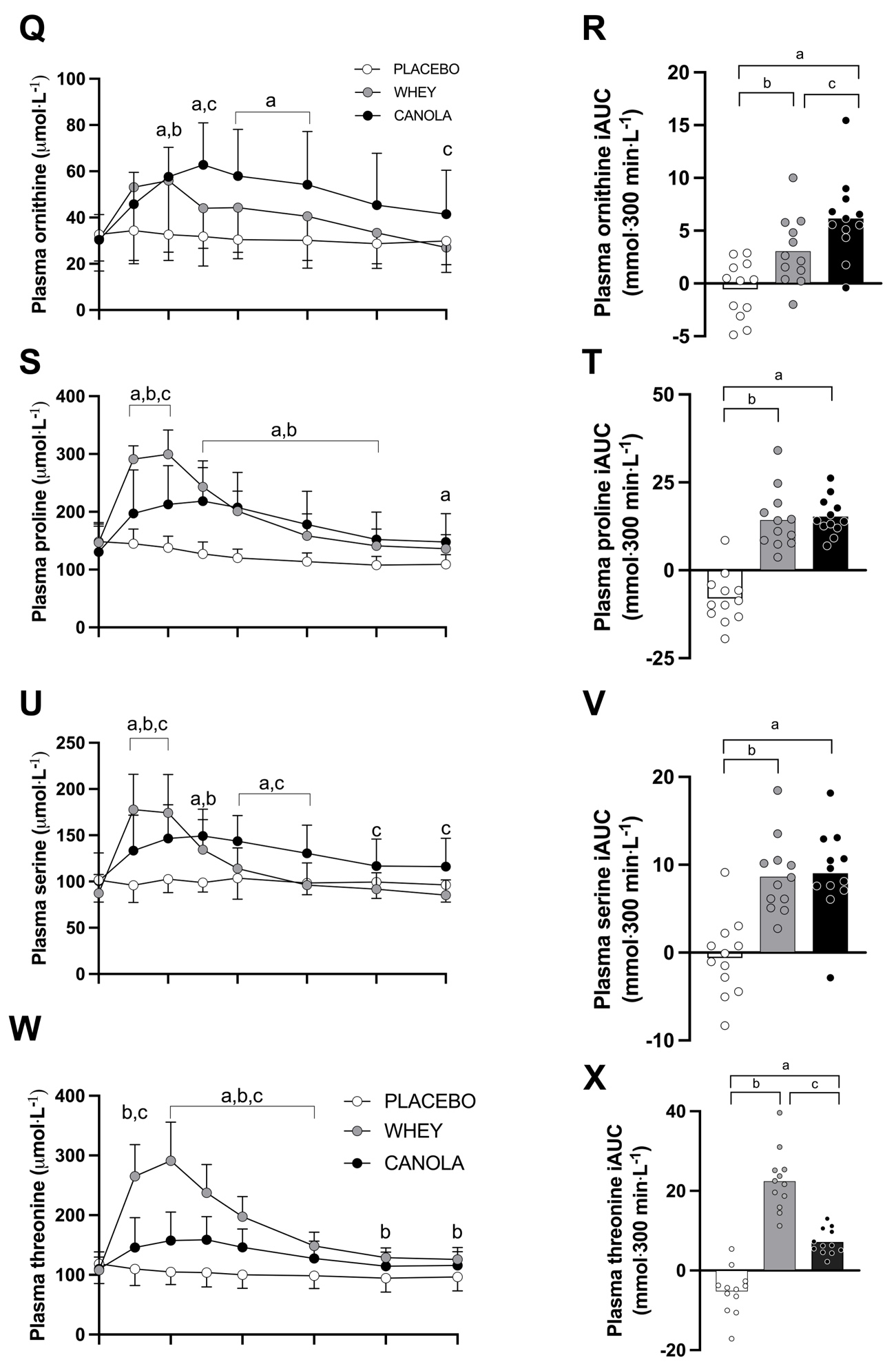


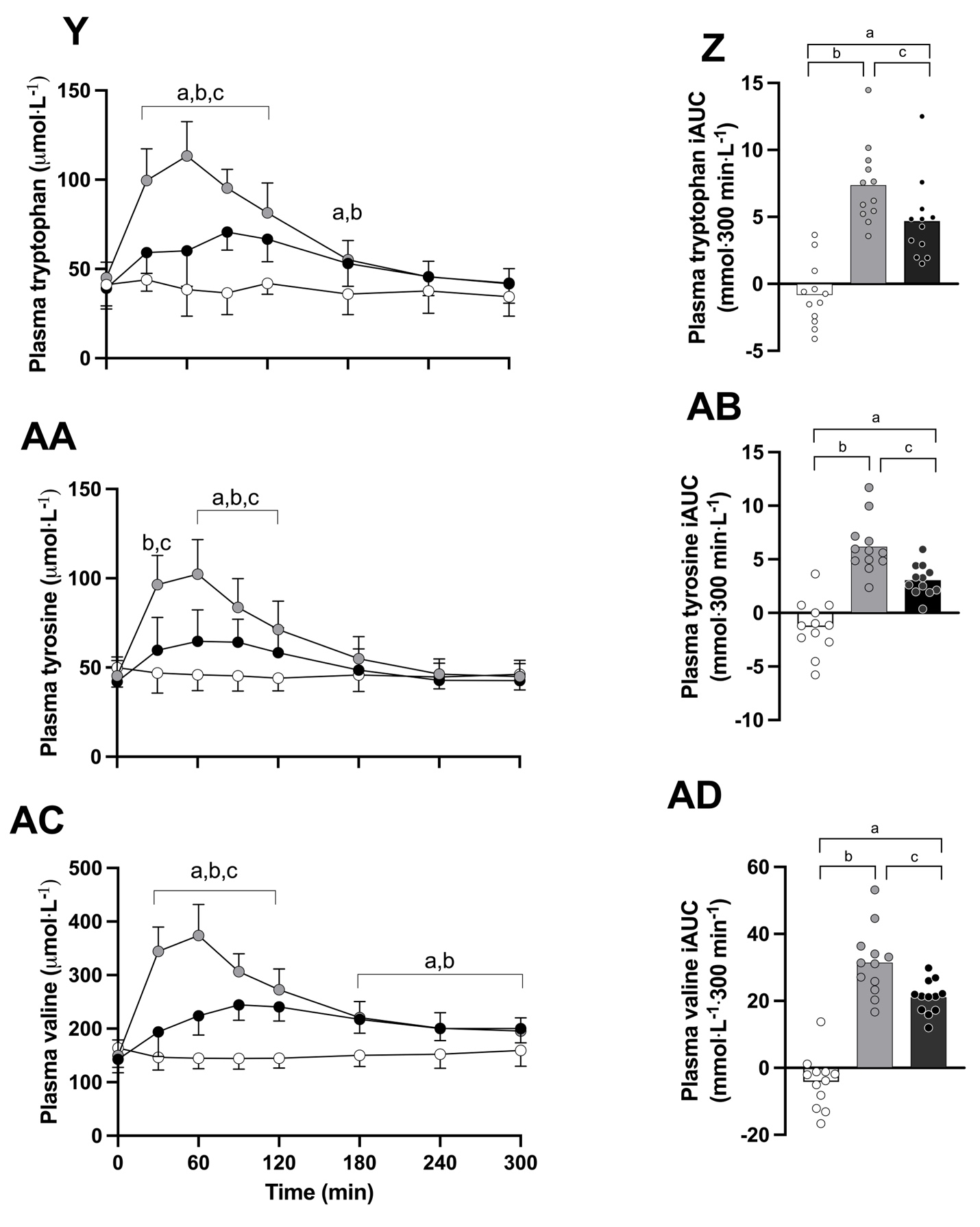


**Supplemental Figure 2.** CONSORT flow diagram. CONSORT: Consolidated Standards of Reporting Trials. Canola: 20 g native canola protein isolate, Whey: 20 g whey protein isolate, Placebo: water.

**Supplemental Figure 3. Anabolic signaling**
Skeletal muscle phosphorylation status (ratio of phosphorylated to total protein) of mTOR (Ser2448) (**A**), p70S6K (Thr389) (**B**), p70S6K (Thr421/Ser424) (**C**), rpS6 (Ser240/244) (**D**), rpS6 (Ser235/236) (**E**) and 4E‐BP1 (Thr37/46) (**F**) in the post-absorptive, basal state (t = -180 min), right after but prior to ingestion of the test beverage (t = 0 min) and 300 min after ingestion of the test beverage (t = 300 min) in healthy, young females (*n=12* per group). Placebo: water, Whey: 20 g whey protein isolate, Canola: 20 g native canola protein isolate. *Significantly different (*P<0.05*) from t = -180 min. *^#^* Significantly different (*P<0.05*) from t = 0 min.

**Supplemental Figure 4. Gene expression**

Skeletal muscle mRNA expression of genes involved in proteolysis: FOXO1 (**A**), MuRF1 (**B**) and MaFBx (**C**) in the post-absorptive, basal state (t = -180 min), right after but prior to ingestion of the test beverage (t = 0 min) and 300-min after ingestion of the test beverage (t = 300 min) in healthy, young females (n=12 per group). Placebo: water, Whey: 20 g whey protein isolate, Canola: 20 g native canola protein isolate. *Significantly different (P<0.05) from t=-180 min. # Significantly different (P<0.05) from t = 0 min.

**SUPPLEMENTAL DATA**

**Mixed-muscle protein synthesis rates, analyzed by two-way repeated measures ANOVA**

A two-way time (basal vs post-prandial) by treatment (Placebo, Canola, Whey) repeated-measures ANOVA revealed a significant main effect of Time, *F*(1, 33) = 64.01, *P<0.001*, η²p = 0.660, indicating increased muscle protein synthesis following resistance with no significant main effect of Treatment *F*(2, 33) = 0.470, *P* = 0.629, η²p = 0.028, and no significant Time × Group interaction, *F*(2, 33) = 1.684, *P*= 0.093, η²p = 0.093.

**Mixed-muscle protein synthesis rates analyzed by two-way repeated measures ANOVA for placebo and combined protein groups**A two-way ANOVA with time (basal vs post-prandial) and treatment (placebo; n = 12 vs. protein n = 24) revealed a significant main effect of Time, *F*(1, 34) = 49.42, P<0.001, η²p =0.592, but no significant main effect of Treatment, *F*(1, 34) = 0.865, *p* = 0.359, η²p = 0.025 or any Time by Treatment interaction, *F*(1, 34) = 2.430, *p* = 0.128, η²p = 0.067.
